# Progress in Medicine

**Published:** 1900-01-06

**Authors:** 


### PROGRESS IN MEDICINE.

### DISEASES OF THE HEART AND CIRCULATION.

(*Continued from page* 211.)

**Infective Endocarditis.**—A discussion[2] on the pathology of infective endocarditis at Portsmouth was opened by Washburn, who said that the disease is an infection of the endocardium by bacteria, which gives rise to necrotic and inflammatory changes. The condition cannot be considered a pathological entity, as several distinct species of bacteria are capable of causing it. The endocardium covering the valves at the points of maximum contact during closure is the portion almost invariably affected. The bacteria may be conveyed to the heart in two ways, either by the blood directly in contact with the endocardium, or by means of small blood vessels in the valves. The absence of vessels precludes the second method of conveyance with previously healthy valves, but with chronically inflamed valves this is possible. The bacterial invasion causes necrosis of the endothelial and sub-endothelial cells, and, as a consequence, a thrombus, consisting at first of blood platelets and later of fibrin and leucocytes, is deposited on the surface, producing the so-called vegetations. After a time proliferation of the sub-endothelial cells occurs, and leucocytes from adjacent blood vessels invade the necrosed tissue and the thrombus. Frequently the necrotic process is in excess of the repair, and ulceration results. The ulceration may spread to the chordæ tendineæ, or to the mural endocardium or intima of the aorta.

Out of 100 cases the right side was affected 14 times, and alone affected in four. The mitral valve was alone affected 38 times, the aortic alone 16 times, and the aortic and mitral together in 32 cases. The cause of the greater preponderance of affection of the left side of the heart is to be found in greater strain on the valves, and on the frequent existence of old, non-infective, endocarditis on that side. In from 60 to 75 per cent. of cases the infective process is engrafted on valves damaged by previous disease, and this is almost invariably on the left side.

The bacteria causing the endocarditis may enter the body through the respiratory, digestive, or genito-urinary tracts, or through wounds in the skin. In about 30 per cent. of cases there is clear evidence of the mode of entrance in some preceding lesion.

The lesions caused by infective endocarditis may be roughly divided into three classes—those due to the mechanical effect of the valvular disorder, such as dropsy, nutmeg liver; those due to toxæmia, such as cloudy degeneration of the liver, enlarged spleen, and acute nephritis; and those due to emboli. Emboli free from bacteria give rise to simple infarcts, those crowded with bacteria to either simple infarcts, or more commonly to suppurative lesions, such as abscesses in the liver, spleen, kidney, and lungs, meningitis, empyema, and aneurisms, owing to softening of the vessel-wall.

The streptococcus pyogenes, the pneumococcus and the staphylococcus aureus, in the order mentioned, are the most common causes of the disease. It is less commonly caused by the gonococcus, and by the tubercle bacillus.

The condition which precedes the endocarditis is often of great assistance in determining the nature of the bacteria causing the disease. For instance, endocarditis following on pneumonia is generally due to the pneumococcus, following on gonorrhœa to the gonococcus. But this is not invariable, for an endocarditis following gonorrhœa may be due to the streptococcus. Examination of the blood during life may reveal the presence of bacteria, but in a large number of cases the examinations have yielded negative results.

Foulerton did not think the incidence of infective endocarditis was markedly favoured by a pre-existing simple endocarditis. One of the reasons why the right side of the heart was often affected was the proximity of the venous system, where septic thrombi were most common, whereas the lungs act as strainers for the left side.

Pakes had examined the bacteriology of 21 cases of infective endocarditis, and had found the streptococcus only 13 times, this and the staphylococcus once, staphylococcus only twice, bacillus pyocyaneus, pneumococcus, and gonococcus each once. In four of the above cases the blood was investigated during life, but with negative results in each case. This was in marked contrast with experience in streptococcal septicæmia, leading to the conclusion that the streptococcus producing infective endocarditis is of much lower virulence than that producing septicæmia, which is borne out by the results of experimental infection of animals by the organisms cultivated from the diseased valves. Abrahams insisted that valvular ulceration is no criterion of the infective nature of an endocarditis; also, emboli may be absent from cases which are in every other way most typical. The spleen is invariably large, even though it contains no infarcts.

Bullock said that the clinical separation of infective from non-infective endocarditis is anything but sharp. Many cases, too, of what one must legitimately diagnose as malignant endocarditis are, as far as present day bacteriological technique goes, unsolved in their etiology. Thus no micro-organisms were found, either during life or after death, in six cases which presented intermittent fever, anæmia, asthenia, and profound endocardial symptoms, and in whom intense verruceous endocarditis was found after death.

Kelynack[3] has analysed the post-mortem records of 20 cases of infective endocarditis. In 15 of the 20 cases there were distinct signs of old valvular disease. The spleen was enlarged in 17, normal in three cases. Infarction was noted in 12 cases, five times in the kidneys, nine times in the spleen, twice in the lung, and once in the brain. According to Ebstein,[4] the earlier, other things being equal, cerebral symptoms appear in infective endocarditis the more rapidly the disease progresses towards a fatal termination. The fever is characterised by wide fluctuations of temperature which follows no constant course. Even numerous chills occurring daily for weeks cannot be looked upon with certainty as a criterion of the presence of metastatic abscesses. It is only possible to diagnose the typhoid form of infective endocarditis in the proved absence of other morbid processes capable of causing a typhoid state independently of infective endocarditis.

An important case has been recorded by Rogers[5] showing that even when a streptococcic infective endocarditis has been definitely diagnosed injection of anti-streptococcic serum does not always cure. The affection followed on rheumatic arthritis and endocarditis, and was proved to be due to streptococci by examination of the blood. Injection of the serum did not have the least beneficial effect. The explanation is probably to be found in the lately-discovered fact that there are several species of streptococcus pyogenes aureus, and that a serum prepared from one species will not protect against disease caused by another species.

[2] Brit. Med. Jour., Nov. 4, 1899. [3] Kelynack, Med. Chron., Aug., 1899. [4] Ebstein, Deut. Archiv. f. Klin. Med. B. 63. [5] Rogers, Lancet, June 10, 1899.

